# RpoS-independent evolution reveals the importance of attenuated cAMP/CRP regulation in high hydrostatic pressure resistance acquisition in *E. coli*

**DOI:** 10.1038/s41598-017-08958-z

**Published:** 2017-08-17

**Authors:** Elisa Gayán, Alexander Cambré, Chris W. Michiels, Abram Aertsen

**Affiliations:** KU Leuven, Laboratory of Food Microbiology, Department of Microbial and Molecular Systems (M2S), Faculty of Bioscience Engineering, Leuven, Belgium

## Abstract

High hydrostatic pressure (HHP) processing is an attractive non-thermal alternative to food pasteurization. Nevertheless, the large inter- and intra-species variations in HHP resistance among foodborne pathogens and the ease by which they can acquire extreme resistance are an issue of increasing concern. Since RpoS activity has been considered as a central determinant in the HHP resistance of *E. coli* and its pathovars, this study probed for the potential of an *E. coli* MG1655 Δ*rpoS* mutant to acquire HHP resistance by directed evolution. Despite the higher initial HHP sensitivity of the Δ*rpoS* mutant compared to the wild-type strain, evolved lineages of the former readily managed to restore or even succeed wild-type levels of resistance. A number of these Δ*rpoS* derivatives were affected in cAMP/CRP regulation, and this could be causally related to their HHP resistance. Subsequent inspection revealed that some of previously isolated HHP-resistant mutants derived from the wild-type strain also incurred a causal decrease in cAMP/CRP regulation. cAMP/CRP attenuated HHP-resistant mutants also exhibited higher resistance to fosfomycin, a preferred treatment for STEC infections. As such, this study reveals attenuation of cAMP/CRP regulation as a relevant and RpoS-independent evolutionary route towards HHP resistance in *E. coli* that coincides with fosfomycin resistance.

## Introduction

High hydrostatic pressure (HHP) treatment is a promising alternative to conventional thermal pasteurization of foods as it is able to improve the microbial stability and safety of foods without compromising its fresh-like attributes. However, while commercial HHP treatment ranging from 400 to 600 MPa can achieve a significant level of reduction of most vegetative spoilage and pathogenic foodborne bacteria^1^, an important concern remains the extensive HHP resistance displayed by some bacterial strains^2–4^. In particular, a substantial proportion of Shiga-toxigenic *Escherichia coli* (STEC) isolates appears to be resistant to exposures of 600 MPa^3, 5^. Furthermore, extreme HHP resistance is a trait that can reproducibly be genetically acquired by *E. coli* when iteratively exposed to HHP shocks^4, 6^.

The mechanism underlying bacterial inactivation by HHP treatment is still not well understood; however, some cellular targets and stress responses involved in pressure survival have been identified^7–9^. In particular, the general stress response governed by the RpoS sigma factor strongly contributes to HHP resistance in *E. coli*, which in part could be traced back to the RpoS mediated upregulation of cyclopropane fatty acid synthase, outer membrane lipoproteins and trehalose-6-phosphate synthase^10, 11^. Indeed, stationary phase induction of this general stress response accounts for the increased HHP resistance of stationary over exponential phase cells^10^. Moreover, also the large variability in pressure resistance among natural STEC isolates has been related to polymorphisms in their *rpoS* allele and the corresponding variations in cellular RpoS activity^5, 11^.

These polymorphisms are thought to reflect the evolutionary adaptation of different *E. coli* populations to their particular ecological niches^12, 13^. In fact, strains with attenuated RpoS activity tend to display an improved use of secondary carbon sources like succinate, fumarate and acetate at the expense of self-preservation under stress^12, 14^, while strains with a high basal RpoS activity display increased stress resistance at the expense of nutritional competence^15^. Furthermore, given the extensive regulatory network surrounding RpoS activity^16^, modulatory mutations affecting this activity are likely to occur at a relative high frequency, enabling lineages to balance between self-preservation (with high RpoS activity) and nutritional competence (with low RpoS activity) (also referred to as the SPANC balance)^17^. As a result, modulation of the RpoS response seems to be an important evolutionary strategy in *E. coli* to improve nutritional competence on the one hand or stress tolerance and cross-resistance on the other.

The latter also became clear when exposure of *E. coli* O157:H7 (ATCC 43888) to a limited number of progressively intensifying HHP shocks rapidly selected for mutants that were cross-resistant to mild pressure (*id est* 300 MPa) and heat, and that showed signs of increased RpoS activity^15^. A random transposon knock-out screen subsequently confirmed the role of RpoS activity in HHP resistance of ATCC 43888, as a disruption of the *rssB* gene (encoding an anti-RpoS factor that binds to RpoS and quenches its activity^16^) yielded a similar HHP and heat cross-resistance^18^. Interestingly, the same transposon screen also revealed that disruption of either the *cyaA* (encoding the adenylate cyclase or cAMP synthetase) or *crp* (encoding the cAMP Receptor Protein) gene could confer HHP resistance as well. Even though lack of cAMP/CRP regulation has been described to increase *rpoS* expression, it remains unclear whether or not the underlying mechanism is dependent on RpoS^18, 19^. Nevertheless, given the growth defect imposed by *cyaA* or *crp* mutations^20^, we anticipated that mutants spontaneously compromised in these loci could not readily enrich in populations evolving towards HHP resistance^18^.

Given the central and perhaps also dominant role of the RpoS global regulator in HHP resistance and resistance development, this study aimed to investigate the remaining potential of *E. coli* to develop HHP resistance in the absence of this sigma factor, in order to elucidate potential alternative evolutionary pathways that are truly independent of RpoS upregulation.

## Results

### Evolution of *E. coli* MG1655 Δ*rpoS* towards HHP resistance

**Figure 1:**
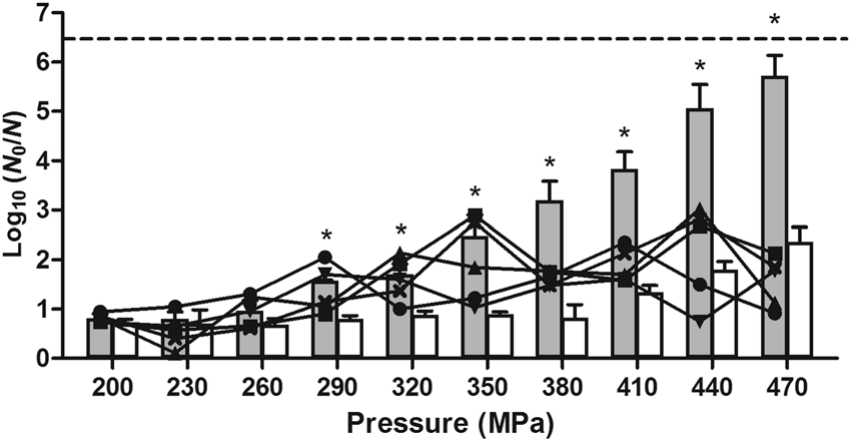
Evolution of the HHP resistance of five independent axenic cultures of an *E. coli* MG1655 ∆*rpoS* strain iteratively exposed to progressively intensifying HHP shocks (15 min) with intermittent resuscitation and growth of survivors. The line graphs show the inactivation (*i.e*. logarithmic reduction factor, log_10_ [*N*_0_/*N*]) of the five evolving lineages (●, lineage 1; ■, lineage 2; ▼, lineage 3; ▲, lineage 4; ×, lineage 5) during the stepwise selection regime with 30 MPa increments, while the gray bars represent the resistance of the unevolved MG1655 ∆*rpoS* parental strain (*i.e*. without previous HHP exposure). To illustrate the effect of RpoS activity on HHP resistance, the inactivation of *E. coli* MG1655 WT (*i.e*. RpoS proficient) strain is included as well (white bars). The dotted line represents the quantification limit (1,000 CFU/ml). An asterisk indicates statistically significant differences (*P* ≤ 0.05) between the inactivation of the parental ∆*rpoS* and WT strain at indicated pressures.

To examine the intrinsic ability of *E. coli* MG1655 lacking RpoS to develop HHP resistance, an MG1655 Δ*rpoS* derivative was constructed and five independent cultures of this mutant strain were subjected to successive cycles of increasingly severe HHP shock (starting at 200 MPa, with 30 MPa increments each cycle up to 470 MPa) with intermittent outgrowth of survivors. Figure 1 illustrates the evolution of the HHP resistance of the Δ*rpoS* lineages during the selection regimen in comparison to the inactivation of the original Δ*rpoS* and WT parental strains. As anticipated, deprivation of the *rpoS* gene sensitized the Δ*rpoS* strain to HHP treatment, resulting in a *circa* 1,000-fold higher (*P* ≤ 0.05) inactivation at 470 MPa compared to the WT strain.

**Figure 2:**
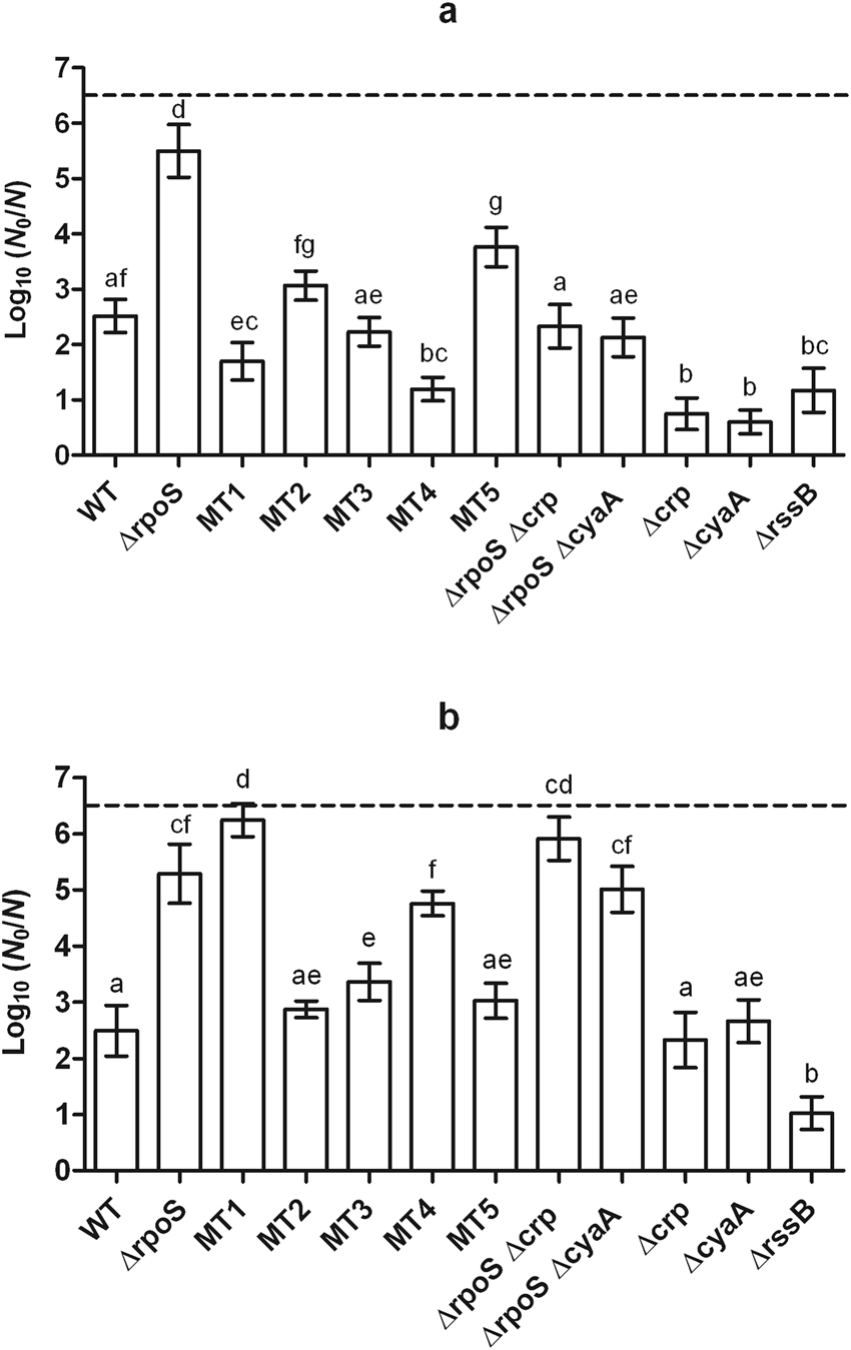
Logarithmic reduction factor of *E. coli* MG1655 WT and indicated mutants by a HHP treatment at 500 MPa (**a**) or heat treatment at 57.0 °C (**b**) for 15 min. The dotted line represents the quantification limit (1,000 CFU/ml). Letters indicate statistically significant differences (*P* ≤ 0.05) among the inactivation of all strains.

Despite this parental HHP hypersensitivity, however, each of the five evolving MG1655 Δ*rpoS* lineages managed to develop piezotolerance, with the single clones subsequently isolated from each of the five lineages (designated MT1 to MT5) being on average 2.8 log_10_ cycles more resistant than the parental ∆*rpoS* background at 470 MPa (data not shown). Further phenotypic analysis of these five clones revealed varying levels of HHP resistance, with three clones even displaying a HHP resistance equal (*P* > 0.05; *i.e*. MT3) or significantly (*P* ≤ 0.05) higher (*i.e*. MT1 and MT4) than the MG1655 WT (*i.e. rpoS* proficient) parent at 500 MPa despite lacking RpoS activity (Fig. 2A). Interestingly, HHP resistance in the evolved ∆*rpoS* strains did not necessarily coincide with heat resistance as MT1 even became significantly (*P* ≤ 0.05) more sensitive to a 57 °C shock than its ∆*rpoS* parent (Fig. 2B).

Importantly, when three independent lineages of the ancestral ∆*rpoS* strain were subcultured for *ca*. 66 generations without imposing any stress, their HHP inactivation (at 470 MPa) actually significantly (*P* ≤ 0.05) increased by 0.7 log_10_ cycle on average (data not shown), indicating that serial passaging itself did not select for pressure resistance.

### Compromised CRP activity can explain the HHP resistance phenotype of some evolved *E. coli* MG1655 Δ*rpoS* mutants

Since a random transposon knock-out screen performed earlier in *E. coli* O157:H7 (strain ATCC 43888) had revealed that disruption of cAMP/CRP homeostasis could confer HHP resistance^18^, we examined whether cAMP/CRP regulation was compromised in the evolved Δ*rpoS* mutants. As lactose metabolism in *E. coli* requires a functional cAMP/CRP complex, we first examined the evolved Δ*rpoS* mutants for their lactose fermentation phenotype on MacConkey agar (Table 1 and Supplementary Fig. S1). Interestingly, the size of the pink halo of the most piezotolerant Δ*rpoS* mutants (*i.e*. MT1, MT3 and MT4) was visibly reduced in comparison to the Δ*rpoS* parent. Moreover, this phenotype persisted regardless of the addition of cAMP to the MacConkey medium. Sequencing subsequently revealed that MT1 incurred an IS2 insertion of *ca*. 1.3 kb length in the *crp* promoter region, while the *crp* gene of MT4 incurred a point mutation resulting in an V184M amino acid substitution in the DNA-binding domain of CRP^21^ (Table 1). Most of the randomly picked clones from lineage 1 and 4 (where MT1 and MT4 stemmed from, respectively) displayed a similarly attenuated MacConkey phenotype, indicating that these variants constituted the majority of the evolved populations.

**Table 1:** Phenotypic characterization of *E. coli* MG1655 WT and its indicated mutants on MacConkey lactose agar with or without 5 mM of cAMP added, and on M9 glucose or lactose agar. The corresponding mutations found in the *crp* or *cyaA* sequence (if any) are indicated.

Strain	Size of the pink halo in MacConkey agar^a^		Growth on M9^b^		*crp*/*cyaA* mutation
	−cAMP	+cAMP	Glucose	Lactose	
WT	++++	++++	++++	++++	
Δ*crp*	−	−	+++	−	
Δ*cyaA*	−	++++	+++	−	
Δ*rpoS*	++++	++++	++++	++++	
Δ*rpoS* Δ*crp*	−	−	+++	−	
Δ*rpoS* Δ*cyaA*	−	++++	+++	−	
MT1	+++	+++	++++	++	*crp*: insertion of IS2 in the promoter region
MT2	++++	++++	++++	++++	
MT3	++	++	++++	++++	
MT4	−	−	+++	+	*crp*: G550A
MT5	++++	++++	++++	++++	
LMM1010	+	++++	++++	++	*cyaA*: insertion of A at position 990
LMM1020	++	++	++++	+++	*crp*: G248C, T446A
LMM1030	++++	++++	++++	++++	
DVL20	++++	++++	++++	++++	
DVL1	+	++++	++++	++	*cyaA*: insertion of TATAG at position 1,923

^a^The size of the pink halo formed by 20 µl of stationary phase cultures diluted 1/100 in 0.85% KCl after overnight incubation was scored from − (no halo) to ++++ with respect to the Δ*crp* and Δ*cyaA* mutants and the WT and ∆*rpoS* parent, respectively (Supplementary Fig. S1).

^b^The growth of 5 µl of stationary phase cultures diluted 1/100 in M9 salts after 48 h of incubation was scored from − (no growth) to ++++ with respect to the Δ*crp* and Δ*cyaA* mutants and the WT and ∆*rpoS* parent, respectively.

Despite the fact that MT3 clearly displayed a reduced pink halo on MacConkey agar, it did not show growth defects on M9 lactose (Table 1) or significantly (*P* > 0.05) reduced β-galactosidase activity compared to its ∆*rpoS* parent as did MT1 and MT4 (Supplementary Fig. S2). Furthermore, its *crp* and *cyaA* loci appeared to be intact, and therefore the altered MacConkey phenotype of this mutant is likely not causally linked to cAMP/CRP downregulation. In MT2 and MT5, growth on MacConckey agar and M9 lactose, β-galactosidase activity and the sequences of *crp* and *cyaA* loci were unaltered compared to the Δ*rpoS* parent (Table 1 and Supplementary Fig. S2).

**Figure 3:**
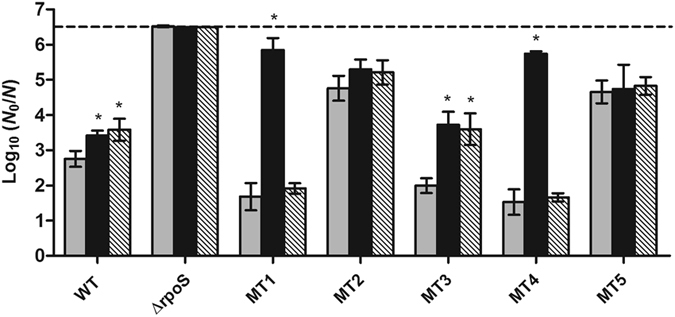
Logarithmic reduction factor of *E. coli* MG1655 WT, Δ*rpoS* and Δ*rpoS* evolved mutants equipped with pACYC184 (control vector; grey bars), pACYC184-*crp* (black bars) or pACYC184-*cyaA* (hatched bars) by a HHP treatment at 500 MPa for 15 min. The dotted line represents the quantification limit (1,000 CFU/ml). An asterisk indicates statistically significant differences (*P* ≤ 0.05) between the inactivation of each strain equipped with pACYC184-*crp* or pACYC184-*cyaA* and the control vector.

In order to confirm that a compromised CRP functionality is indeed causally linked to the HHP resistance phenotype of MT1 and MT4, the evolved Δ*rpoS* mutants were equipped with a plasmid-borne copy of the *crp* gene (using pACYC184-*crp* ^18^) and exposed to pressure (500 MPa). Please note that the HHP resistance of the ∆*rpoS* parent and some of its derivatives (*i.e*. MT2 and MT5) significantly (*P* ≤ 0.05) decreased by *ca*. 1.1 log_10_ cycles in the presence of the pACYC184 control vector (Figs. 2A and 3), likely due to energy draining that can constitute a burden for the cells^22^. To avoid the interference of this artefact in the interpretation of the complementation assays, the effect of gene complementation on HHP survival was evaluated in comparison to the strains equipped with the control plasmid. As shown in Fig. 3, provision of CRP significantly (*P* ≤ 0.05) decreased the HHP survival of MT1 and MT4 by 4.2 log_10_ cycles on average. Moreover, in agreement with the lack of cAMP complementation already observed in the lactose fermentation assay (Table 1), complementation with a plasmid-borne *cyaA* gene (using pACYC184-*cyaA* ^18^) did not significantly (*P* > 0.05) attenuate the HHP resistance of MT1 and MT4. The inactivation of the ∆*rpoS* parent also decreased significantly (*P* ≤ 0.05) but to a lower extent (*ca*. 0.8 log_10_ cycle) when equipped with pACYC184-*crp* and pACYC184-*cyaA* at 400 MPa (Supplementary Fig. 3S). In contrast, neither of the complementation plasmids significantly (*P* > 0.05) affected the HHP resistance of MT2 and MT5. In the case of MT3, which displayed altered MacConkey phenotype without compromising lactose fermentation and harboring intact *crp* and *cyaA* alleles, complementation with both pACYC184-*crp* or pACYC184-*cyaA* appeared to significantly (*P* ≤ 0.05) attenuate HHP resistance by 1.7 log_10_ cycles on average, indicating that this mutant might for some other reason be more susceptible to overdosing CRP and CyaA.

### Compromised cAMP/CRP regulation also contributes to the phenotype of extremely HHP resistant *E. coli* MG1655 mutants

Spurred by the impact and evolvability of cAMP/CRP regulation in HHP resistance development of the Δ*rpoS* parent, a number of previously documented mutants that were evolved starting from a wild-type (*i.e*. RpoS proficient) MG1655 background to withstand exposure to 800 MPa and higher^4, 6, 23^ were evaluated on MacConkey agar, M9 lactose and β-galactosidase activity for their capacity to utilize lactose (Table 1 and Supplementary Fig. S2).

**Figure 4:**
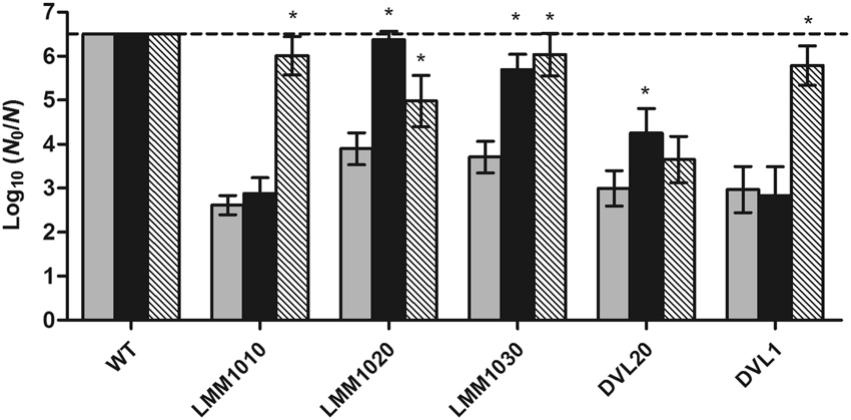
Logarithmic reduction factor of *E. coli* MG1655 WT and its evolved mutants equipped with pACYC184 (control vector; grey bars), pACYC184-*crp* (black bars) or pACYC184-*cyaA* (hatched bars) by a HHP treatment at 800 MPa for 15 min. The dotted line represents the quantification limit (1,000 CFU/ml). An asterisk indicates statistically significant differences (*P* ≤ 0.05) between the inactivation of each strain equipped with pACYC184-*crp* or pACYC184-*cyaA* and the control vector.

As such, it appeared that strain LMM1010, LMM1020 and DVL1 indeed displayed a clearly compromised lactose fermentation, and that the addition of cAMP to the MacConkey medium could completely alleviate this phenotype in LMM1010 and DVL1. Correspondingly, LMM1010 and DVL1 were subsequently found to harbor frameshift mutations in their *cyaA* gene, while LMM1020 incurred two point mutations in its *crp* gene (Table 1). The latter resulted in two amino acid substitutions, one in the cAMP binding site (R83P) and another in the DNA binding domain (L149Q)^21^. In agreement with the observed mutations, the HHP resistance of LMM1010 and DVL1 became significantly (*P* ≤ 0.05) attenuated by 3.1 log_10_ reductions on average when equipped with pACYC184-*cyaA* but not with pACYC184-*crp* (Fig. 4). The HHP resistance of LMM1020 was significantly (*P* ≤ 0.05) reduced by 3.0 log_10_ cycles in the presence of pACYC184-*crp* and to a lesser extent (*ca*. 1 log_10_ cycle) by pACYC184-*cyaA*.

Strain LMM1030 and DVL20 did not suffer compromised lactose fermentation nor mutations in their *crp* and *cyaA* loci (Table 1 and Supplementary Fig. S2). The HHP resistance of DVL20 suffered a marginal but significant (*P* ≤ 0.05) reduction of 1.2 log_10_ cycles from the presence of pACYC184-*crp*. Similarly, the survival of the WT strain to 600 MPa also significantly (*P* ≤ 0.05) decreased by 0.9 log_10_ cycle when complemented with both plasmids (Supplementary Fig. S3). Interestingly, both complementation vectors significantly (*P* ≤ 0.05) attenuated the HHP resistance of LMM1030 by 2.3 log_10_ reductions on average, suggesting again that this mutant is sensitized to cAMP/CRP upregulation by an alternative mechanism.

### Synthetic reconstruction confirms lack of cAMP/CRP regulation as cause of HHP resistance in *E. coli* MG1655

**Figure 5:**
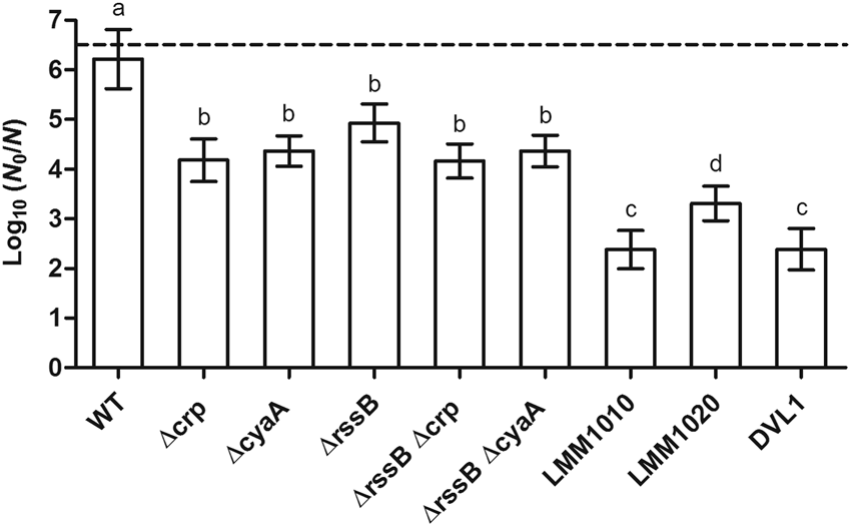
Logarithmic reduction factor of *E. coli* MG1655 WT and indicated mutants by a HHP treatment at 800 MPa for 15 min. The dotted line represents the quantification limit (1, 000 CFU/ml). Letters indicate statistically significant differences (*P* ≤ 0.05) among the inactivation of all strains.

In order to study the impact of cAMP/CRP regulation on HHP resistance in a more defined genetic background (*i.e*. not exposed to directed evolution), the *crp* or *cyaA* gene was synthetically deleted in the WT or Δ*rpoS* parent, after which HHP resistance of the resulting strains was examined. This unambiguously established that complete loss of either of the components giving rise to the cAMP/CRP complex could significantly (*P* ≤ 0.05) improve the HHP resistance in both a Δ*rpoS* (*ca*. 2,000-fold at 500 MPa [Fig. 2A]) and WT (*ca*. 100-fold at 500 MPa [Fig. 2A] and 800 MPa [Fig. 5]) background.

**Figure 6:**
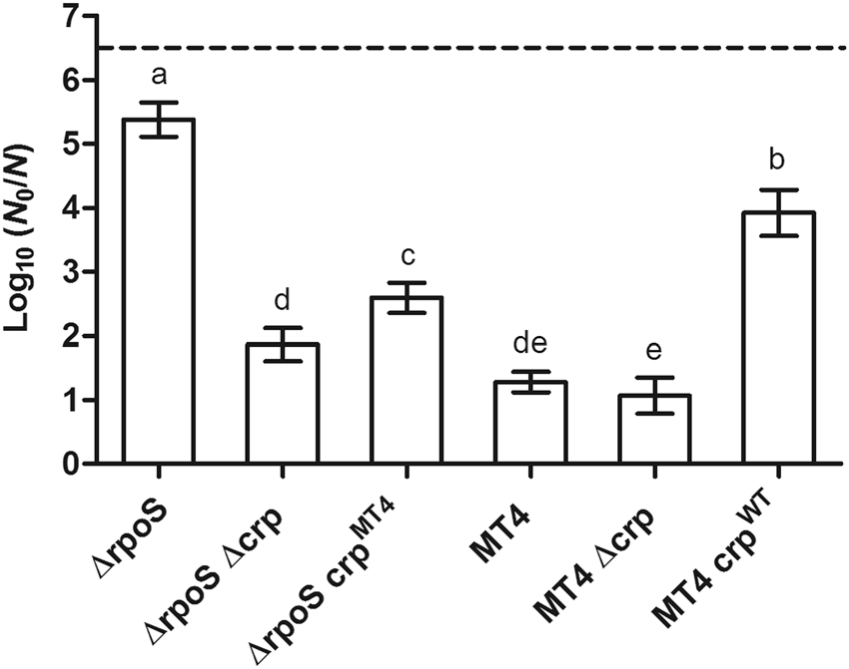
Logarithmic reduction factor of *E. coli* MG1655 ∆*rpoS* equipped with a *crp* deletion (∆*rpoS* ∆*crp*) or the *crp* allele from MT4 (∆*rpoS crp* ^*MT4*^), and MT4 equipped with a *crp* deletion (MT4 ∆*crp*) or the wild-type *crp* allele (MT4 *crp* ^*WT*^) by a HHP treatment at 500 MPa for 15 min. The dotted line represents the quantification limit (1,000 CFU/ml). Letters indicate statistically significant differences (*P* ≤ 0.05) among the inactivation of all strains.

Furthermore, when the *crp* allele of MT4 (*i.e*. the most HHP resistant mutant obtained in the ∆*rpoS* parent) was chromosomally reconstructed in its parental strain (leading to Δ*rpoS crp* ^*MT4*^), the HHP resistance of ∆*rpoS* strain increased *ca*. 600-fold (*P* ≤ 0.05) at 500 MPa (Fig. 6). In the same vein, replacing the chromosomal *crp* ^*MT4*^ allele in MT4 with the fully functional wild-type *crp* allele (leading to MT4 *crp* ^*WT*^) significantly (*P* ≤ 0.05) attenuated the HHP resistance of MT4 by 2.6 log_10_ cycles, while similarly replacing the *crp* ^*MT4*^ allele with the non-functional Δ*crp* allele (leading to MT4 Δ*crp*) did not affect (*P* > 0.05) its HHP resistance (Fig. 6). These observations further underscore the central role of *crp* ^*MT4*^ as a loss-of-function allele in the HHP resistance of MT4.

It should nevertheless be noted that the HHP resistance of the spontaneously evolved cAMP/CRP compromised mutants (*i.e*. obtained after directed evolution of the Δ*rpoS* and WT parents) often still remained higher than the synthetically reconstructed strains, suggesting secondary determinants to contribute to HHP resistance as well. In particular, while the survival of MT1 to 500 MPa matched (*P* > 0.05) the inactivation of the Δ*rpoS* background lacking *crp* or *cyaA* locus, the HHP resistance of MT4 was *ca*. 1 log_10_ cycle significantly (*P* ≤ 0.05) higher than that of Δ*rpoS* Δ*crp* and Δ*rpoS* Δ*cyaA* strains (Fig. 2A). Likewise, the synthetically converted MT4 *crp* ^*WT*^ strain clearly suffered attenuated HHP resistance compared to MT4, but still remained *ca*. 30-fold more resistant (*P* ≤ 0.05) than the ∆*rpoS* parent (Fig. 6). Finally, the survival of LMM1010, LMM1020 and DVL1 to extreme HHP treatment (*i.e*. 800 MPa) was also significantly (*P* ≤ 0.05) higher (from 0.9 to 1.9 log_10_ cycles) than that of the WT parent in the absence of *crp* or *cyaA* (Fig. 5).

### HHP resistant *E. coli* MG1655 mutants compromised in cAMP/CRP regulation display cross-resistance to fosfomycin but not to heat

In contrast to its effect on HHP resistance, deletion of *crp* or *cyaA* did not significantly (*P* > 0.05) improve heat resistance in an Δ*rpoS* or WT MG1655 background (Fig. 2B). This specific impact was quite different from an RpoS upregulated mutant such as MG1655 *∆rssB* (which lacks the anti-RpoS factor that would otherwise quench RpoS activity^16, 18^) in which the stimulated general stress response yielded cross-resistance to both HHP and heat (Fig. 2). Since RpoS activity and lack of cAMP/CRP regulation seem to contribute to HHP resistance independently from each other, we examined whether the combination of RpoS upregulation (by deleting the *rssB* gene) and cAMP/CRP downregulation (by also deleting either the *crp* or *cyaA* gene) could further improve HHP resistance. However, it appeared that deletion of *rssB* did not significantly (*P* > 0.05) change the HHP resistance of a Δ*crp* or Δ*cyaA* mutant at 800 MPa (Fig. 5), being all equally *ca*. 100-fold more resistant (*P* ≤ 0.05) than the WT strain.

As it was previously found that mutants with decreased cAMP/CRP activity display an increased resistance to fosfomycin^24, 25^, which has been regarded as one of the preferred antibiotic treatment options for STEC infections^26, 27^, the spontaneous HHP resistant mutants raised in this study were examined for their fosfomycin resistance. Table 2 indeed shows that the fosfomycin MIC value for the mutants directly affected in cAMP/CRP regulation (MT1, MT4, LMM1010, LMM1020 and DVL1; with ∆*crp* and ∆*cyaA* strains as a control) increased from 2-fold (LMM1020 and DVL1) to 16-fold (MT4) compared to their parental strain, while mutants that did not incur *cyaA* or *crp* mutations (MT2, MT3, MT5, LMM1030 and DVL20) maintained the same fosfomycin resistance as their parental strain.

**Table 2:** Fosfomycin MICs obtained for *E. coli* MG1655 WT and its indicated mutants.

Strain	MIC (mg/l)
WT	64
Δ*crp*	1,024
Δ*cyaA*	1,024
Δ*rpoS*	64
Δ*rpoS* Δ*crp*	1,024
Δ*rpoS* Δ*cyaA*	1,024
MT1	256
MT2	64
MT3	64
MT4	1,024
MT5	64
LMM1010	256
LMM1020	128
LMM1030	64
DVL20	64
DVL1	128

## Discussion

RpoS activity has previously been established as a central determinant in stationary phase HHP resistance in *E. coli*, and mutations upregulating the activity of this sigma factor therefore highlight one evolutionary strategy towards HHP resistance development^11, 15^. Surprisingly, however, an *E. coli* genetic background deprived of the RpoS protein and therefore hypersensitive to HHP shock readily managed to acquire wild-type (or even higher) levels of HHP resistance upon recurrent exposure to HHP shock. Further analysis subsequently revealed that compromised cAMP/CRP regulation was causally involved in HHP resistance development of two (*i.e*. MT1 and MT4) of the five independently evolved Δ*rpoS* mutants. Furthermore, mutations compromising cAMP/CRP regulation were also discovered to causally contribute to the phenotype of extremely HHP resistant (and RpoS proficient) *E. coli* mutants that were evolved in earlier studies^6, 23^. To the best of our knowledge, this study is therefore the first to identify and phenotypically validate *cyaA* and *crp* mutations as causal HHP resistance conferring mutations arising in spontaneously evolved mutants of *E. coli*.

In *Listeria monocytogenes* (strains LO28 and ScottA), spontaneous mutations leading to HHP resistance have been documented earlier and found to compromise the *ctsR* gene, which encodes the class III heat shock repressor^28–30^. Disruption of the *ctsR* gene can lead to a defect in the repression of *clpC* family chaperones which results in transcription of these stress response genes and consequently in increased cross-resistance to HHP, heat, acidity and H_2_O_2_^2, 28^. In contrast, cAMP/CRP regulation has a more pleiotropic and metabolically oriented impact on *E. coli* physiology, and a large number of genes are responsive to cAMP/CRP activation or repression^31^. Intriguingly, mutations attenuating CyaA or CRP functionality have previously been found to affect osmotic^32^, acid^19, 33^, heat^34^ and oxidative^19, 35, 36^ stress resistance of *E. coli*, although the latter phenotypes might indirectly stem from upregulation of the *rpoS* gene which is repressed by cAMP/CRP^19, 36^. Moreover, while this report clearly underscores compromised cAMP/CRP activity to support HHP resistance in an RpoS independent manner, both transcriptional regulators oppositely affect a largely overlapping set of downstream genes^37, 38^. As such, it could well be that (some of) the functions required to withstand or address HHP stress in the cell are independently but similarly directed by either the upregulation of RpoS or the downregulation of cAMP/CRP. However, the phenotypic constitution of mutants with increased RpoS activity or decreased cAMP/CRP activity is bound to differ, since RpoS upregulation (by deleting Δ*rssB*) supports both HHP and heat resistance while cAMP/CRP downregulation (by deleting *cyaA* or *crp*) only seems to support HHP resistance in *E. coli* MG1655.

Importantly, we also demonstrated that HHP resistant clones with mutations in *crp* or *cyaA* displayed 2- to 16-fold higher resistance to fosfomycin than the parental strains. Fosfomycin is an antibiotic that targets the UDP-N-acetylglucosamine enolpyruvyl transferase (MurA) that is involved in the first step of peptidoglycan synthesis^39^, and the observed resistance is likely due to decreased expression of the glycerol-3-phosphate transporter (GlpT) and the hexose phosphate transporter (UhpT) in the absence of cAMP/CRP complex^24, 25^. These transporters are responsible for the uptake of fosfomycin in the cell due to its similar structure to phosphorylated sugars^39^. Besides being an alternative drug for urinary and systemic infections caused by multidrug-resistant *Enterobacteriaceae* ^40^, fosfomycin is actually proposed as one of the antibiotic therapies of choice for STEC infections^26, 27^. Indeed, in contrast to DNA damaging antibiotics such as fluoroquinolones and trimethoprim-sulfamethoxazole that tend to activate the lytic cycle of the Shigatoxin (Stx) encoding prophages in STEC and thereby improve the production and release of Stx, fosfomycin has no effect on Stx expression and might therefore reduce the risk of haemolytic uremic syndrome development caused by Stx^41–43^. The potential of HHP treatment to select for cAMP/CRP compromised HHP resistant *E. coli* mutants with a concomitantly decreased susceptibility to fosfomycin can therefore pose a concern for food safety that should be further investigated.

Finally, it should be noted that some ∆*rpoS* mutants developed HHP resistance without apparent *cyaA* or *crp* mutations or lactose metabolism defects (*i.e*. MT2, MT3 and MT5), suggesting still alternative RpoS (and likely cAMP/CRP) independent evolutionary routes towards HHP resistance development exist. Nevertheless, these mutants might still have acquired mutations that directly affect the same downstream effector genes that are otherwise governed by RpoS upregulation or cAMP/CRP downregulation.

In summary, this study (i) highlights attenuation of cAMP/CRP regulation as a novel and valid evolutionary route and an RpoS independent mechanism towards HHP resistance development in *E. coli*, and (ii) draws attention to the decreased fosfomycin susceptibility that tends to coincide with this route of HHP resistance development.

## Methods

### Bacterial strains and growth conditions

*E. coli* K-12 MG1655^44^ and its derivatives listed in Supplementary Table S1 were used throughout this study. The ∆*rpoS* knock-out of MG1655 was constructed by transferring the kanamycin resistance cassette replacing the *rpoS* locus of *E. coli* JW5437^45^ by P1-transduction^46^. Further deletions of *rssB*, *crp* and *cyaA* loci were performed according to the method of Datsenko and Wanner^47^, using the cells of interest equipped with the plasmid pKD46 (encoding λ red recombinase genes behind the *araBAD* promoter) and an amplicon prepared on pKD13 (containing the kanamycin resistance cassette) using the primers listed in Baba *et al*.^45^. The antibiotic marker was flanked by FRT sites to be further excised by transiently equipping the strain with the plasmid pCP20 (expressing the Flp site-specific recombinase^48^), resulting in the desired deletion mutant.

The *crp* alleles were reconstructed by the dual counter-selection system described by Li *et al*.^49^. Firstly, the *crp* gene was replaced by an amplicon containing the *tetA*-*sacB* marker prepared on *E. coli* XTL298 using the primers: 5′-CTACCAGGTAACGCGCCACTCCGACGGGATTAACGAGTGCCGTAAACGACATCAAAGGGAAAACTGTCCATATGC-3′ and 5′-GGCGTTATCTGGCTCTGGAGAAAGCTTATAACAGAGGATAACCGCGCATGTCCTAATTTTTGTTGACACTCTATC-3′. In a second step, counter-selection was used to replace the *tetA*-*sacB* cassette with a *crp* amplicon obtained by the following primers: 5′-ACCCACTTCACTCGCGCTT-3′ and 5′-GGAACGAGGGAGAAGAGCAG-3′. Constructs were verified by PCR and sequencing using the locus specific primers described below.

Where indicated, the strains were transformed with pACYC184-*crp* (encoding the *E. coli* ATCC 43888 *crp* gene under the control of its native promoter^18^), pACYC184-*cyaA* (encoding the *E. coli* ATCC 43888 *cyaA* gene under the control of its native promoter^18^) or the corresponding backbone control plasmid (pACYC184^50^) by electroporation. Please note that while *crp* and *cyaA* sequences originated from *E. coli* ATCC 43888, their sequence identities with the corresponding sequences of *E. coli* MG1655 are 99.5% and 98.2%, respectively, and both pACYC184-*crp* and pACYC184-*cyaA* proved to specifically attenuate the HHP resistance of ∆*crp* and ∆*cyaA* strains, respectively, in both wild-type (WT) and ∆*rpoS* backgrounds (Supplementary Fig. S3).

For strain construction, Lysogeny Broth (LB) medium^51^ was used and when necessary, a final concentration of 50 μg/ml of kanamycin (Panreac-AppliChem, Darmstadt, Germany), 20 μg/ml of tetracycline (Sigma-Aldrich, St. Louis, MO, USA), 100 μg/ml of ampicillin (Fischer Scientific, Pittsburgh, PA, USA) or 30 μg/ml of chloramphenicol (Sigma-Aldrich) was added to select for the presence of recombined amplicons^47, 49^, pKD46 and pCP20^47, 48^ or pACYC184-based vectors^18, 50^, respectively. For inactivation experiments, stationary phase cultures were obtained by inoculating test tubes containing 4 ml of Tryptone Soy Broth (TSB; Oxoid, Basingstoke, UK) with a single colony of the required strain prior to incubating the culture aerobically with shaking (300 rpm) for 18 h at 37 °C. In pACYC184-based complementation assays, 30 μg/ml of chloramphenicol supplemented TSB was used to select for the presence of the plasmids^18, 50^.

### HHP and heat treatment

Cells from a stationary phase culture, containing *ca*. 2 × 10^9^ Colony Forming Units per milliliter (CFU/ml), were harvested by centrifugation (4000 × *g*, 5 min) and resuspended in an equal volume of 0.85% KCl (Sigma-Aldrich). For HHP treatment, 200 μl of the suspension was heat sealed in a sterile polyethylene bag after exclusion of the air bubbles and subjected to pressure (200–800 MPa) for 15 min in an 8-ml pressure vessel (HPIU-10000, 95/1994; Resato, Roden, The Netherlands), held at 20 °C with an external water jacket connected to a cryostat. Both the slow pressure increase (100 MPa/min) and the external water jacket attenuated adiabatic heating during pressure build-up. Finally, decompression was almost instantaneous. For heat treatment, on the other hand, three sterile PCR tubes were aseptically filled with a 75 μl portion of resuspended cells and subjected to 57.0 °C for 15 min using a PCR apparatus (Tpersonal 48, Biometra GmbH, Goettingen, Germany). The intensity of HHP and heat treatment was chosen to inactivate ≥ 5 log_10_ reductions of the WT (800 MPa) or ∆*rpoS* (500 MPa; 57 °C) parental strain for a 15 min treatment in order to clearly observe levels of HHP and heat resistance development in the mutants and plasmid complementation effects. After HHP or heat treatment, samples were aseptically retrieved from the polyethylene bags or PCR tubes, respectively, and survival was determined as described below.

### Selection of HHP-resistant mutants by directed evolution

To obtain *E. coli* MG1655 Δ*rpoS* mutants with enhanced pressure resistance, five independent cultures of this strain were reiteratively exposed to HHP shocks (15 min), progressively increasing pressure by 30 MPa each cycle (from 200 MPa to 470 MPa). After each HHP shock, an aliquot of the treated sample was inoculated 1/100 into fresh, prewarmed TSB and regrown for 23 h at 37 °C prior to the next round of pressurization. After ten cycles of selection, two surviving clones from each of the evolved cultures were purified on TSA and rechallenged to the last HHP treatment (470 MPa) in order to select a clone that represented the HHP resistance of the evolved culture. Simultaneously, in order to determine the effect of the serial passage itself in the selection of *E. coli* HHP resistance, three independent lineages of the parental Δ*rpoS* strain were daily subcultured in the absence of HHP stress, which corresponded to *ca*. 6.6 ( = log_2_ 100) generations per day.

### Determination of viability

Samples were serially diluted in 0.85% KCl supplemented with 0.1% bacteriological peptone water (Oxoid), and subsequently 5 μl-samples of each dilution were spotted onto Tryptone Soy Agar (TSA; Oxoid), as previously described^52^. After 24 h of incubation at 37 °C, spots containing between 5–50 colonies were counted, so that the quantification limit was 1,000 CFU/ml. The logarithmic reduction factor was calculated as log_10_ (*N*_0_/*N*), in which *N*_0_ and *N* represent the number of survivors in CFU/ml prior and after treatment, respectively.

### Evaluation of cAMP/CRP downregulation in *E. coli* mutants

The functionality of cAMP/CRP regulation in *E. coli* mutants was screened indirectly by their ability to ferment lactose in MacConkey agar No 3 (Lab M, Lancashire, UK). A spot of 20 µl of stationary phase cultures diluted 1/100 in 0.85% KCl was plated on the MacConkey agar supplemented with or without 5 mM of 3′,5′-cyclic adenosine monophosphate (cAMP; Calbiochem, Darmstadt, Germany), and then incubated overnight at 37 °C. *E. coli* forms pink colonies and a hazy precipitate of bile salts because of acidification^53^. Strains lacking *crp* or *cyaA* locus, and therefore compromised in lactose fermentation, form yellowish colonies because of ammonia production^19^. The addition of exogenous cAMP to MacConkey agar reverts the phenotype of Δ*cyaA* strains to the WT strain (Table 1).

To determine the ability of growth on glucose or lactose as the sole carbon source, stationary phase cultures were washed twice and diluted 1/100 in M9 salts^51^. Subsequently, 5 μl of this suspension was spotted on M9 agar supplemented with either D-glucose (0.4%; Sigma-Aldrich) or D-lactose (0.4%; Acros Organics, Morris Plains, NJ, USA) and incubated at 37 °C for 48 h.

The β-galactosidase assay was carried out as previously described by Miller^51^. Cells were grown in LB in the presence of 1 mM isopropyl β-D-1-thiogalactopyranoside (IPTG; Acros Organics) to mid-exponential phase. The β-galactosidase activity of permeabilized cells on ortho-nitrophenyl-β-galactoside (ONPG; Acros Organics) cleavage was measured in a Multiskan RC (Thermo Labsystems, Vantaa, Finland) and expressed in Miller units (MU).

### Sequencing of *rpoS*, *rssB*, *crp* and *cyaA* loci of *E. coli* mutants

A PCR amplicon encompassing the open reading frame and its upstream promoter region of the *E. coli* MG1655 *rpoS*, *rssB*, *crp* and *cyaA* loci was generated using the site-specific primers previously described^15, 18^ and sequenced by Macrogen (Amsterdam, The Netherlands). The sequences were compared with those from the parental strain (WT or ∆*rpoS* background), which did not differ from the published genome of *E. coli* str. K-12 substr. MG1655 in the GenBank database^44^.

### Fosfomycin susceptibility test

The minimal inhibitory concentration (MIC) for fosfomycin was determined by the broth microdilution method described by Jorgensen and Ferraro^54^. Serial twofold dilutions (1–4,096 mg/l) of fosfomycin (Sigma-Aldrich) in TSB were prepared in a 96-well plate and inoculated with 5 × 10^5^ CFU/ml of the corresponding *E. coli* mutant. The inoculated plate was incubated at 37 °C overnight. The MIC of fosfomycin was defined as the lowest concentration that inhibited visible growth of the corresponding mutant. MIC value for each strain was obtained in triplicate.

### Statistical analysis

Statistical analyses, ANOVA and t-test, were carried out using the software GraphPad PRISM 5.0 (GraphPad Software Inc., San Diego, CA, USA), and differences were regarded as significant when *P* was ≤ 0.05. All microbial inactivation outcomes shown in figures correspond to averages and standard deviations calculated from three replicates performed in different working days. Miller units data correspond to averages and standard deviations obtained in three independent measurements.

## Electronic supplementary material

Supplementary Information
