# Supplementary material for: RpoS-independent evolution reveals the importance of attenuated cAMP/CRP regulation in high hydrostatic pressure resistance acquisition in E. coli
Source: Sci Rep. 2017 Aug 17;7:8600. doi: 10.1038/s41598-017-08958-z (PMC5561100; doi:10.1038/s41598-017-08958-z)
Supplement: Supplementary file 1 — Supplementary Information [file 41598_2017_8958_MOESM1_ESM.pdf]

RpoS-independent evolution reveals the importance of attenuated cAMP/CRP regulation in high hydrostatic pressure resistance acquisition in *E. coli*

Elisa Gayán, Alexander Cambré, Chris W. Michiels, Abram Aertsen*

Supplementary Table S1. Bacterial strains and plasmids used in this study.

Bacteria and plasmids	Description	Source or reference
Escherichia coli		
MG1655 (WT)	Parental wild-type strain	44
MG1655 Δ*crp*	MG1655 carrying in frame deletion of *crp*	This study
MG1655 Δ*cyaA*	MG1655 carrying in frame deletion of *cyaA*	This study
MG1655 Δ*rpoS*	MG1655 carrying in frame deletion of *rpoS*	This study
MG1655 Δ*rpoS* Δ*crp*	MG1655 carrying in frame deletion of *rpoS* and *crp*	This study
MG1655 Δ*rpoS* crp^{MT4}	MG1655 carrying in frame deletion of *rpoS* and the *crp* allele of MT4	This study
MG1655 Δ*rpoS* Δ*cyaA*	MG1655 carrying in frame deletion of *rpoS* and *cyaA*	This study
MG1655 Δ*rssB*	MG1655 carrying in frame deletion of *rssB*	This study
MG1655 Δ*rssB* Δ*crp*	MG1655 carrying in frame deletion of *rssB* and *crp*	This study
MG1655 Δ*rssB* Δ*cyaA*	MG1655 carrying in frame deletion of *rssB* and *cyaA*	This study
LMM1010	HHP resistant derivative of MG1655 (WT)	23
LMM1020	HHP resistant derivative of MG1655 (WT)	23
LMM1030	HHP resistant derivative of MG1655 (WT)	23
DVL20	HHP resistant derivative of MG1655 (WT)	4
DVL1	HHP resistant derivative of MG1655 (WT)	6
MT1	HHP resistant derivative of MG1655 Δ*rpoS*	This study
MT2	HHP resistant derivative of MG1655 Δ*rpoS*	This study
MT3	HHP resistant derivative of MG1655 Δ*rpoS*	This study
MT4	HHP resistant derivative of MG1655 Δ*rpoS*	This study
MT4 Δ*crp*	MT4 carrying in frame deletion of *crp*	This study
MT4 crp^{WT}	MT4 carrying the *crp* allele of MG1655 (WT)	This study
MT5	HHP resistant derivative of MG1655 Δ*rpoS*	This study
Plasmids		
pKD46	Expression of γ, β and exo recombination genes of phage λ under the control of *araBAD* promoter, temperature-sensitive, Ap^R	47
pKD13	Template plasmid containing *kan* gene flanked by FRT sites, Ap^R and Km^R	47
pCP20	Expression of Flp recombinase, temperature-sensitive, Ap^R and Cm^R	48
pACYC184	Cloning vector, Tc^R and Cm^R	50
pACYC184-*crp*	*crp* gene and promoter region of *E. coli* ATCC 43888 cloned in pACYC184, Cm^R	18
pACYC184-*cyaA*	*cyaA* gene and promoter region of *E. coli* ATCC 43888 cloned in pACYC184, Cm^R	18

Supplementary Figure S1

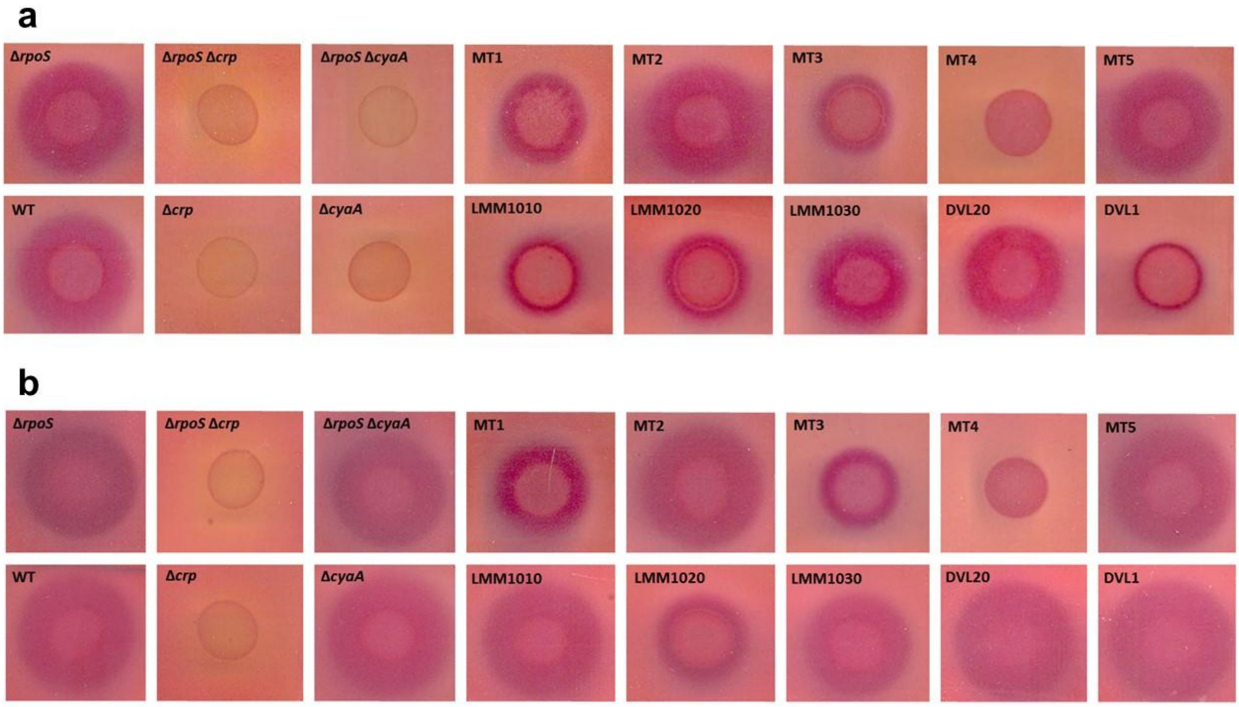

Growth of *E. coli* MG1655 WT, Δ*rpoS* and their evolved mutants on MacConkey lactose agar without (a) or with (b) 5 mM of cAMP added. The strains lacking *crp* or *cyaA* were included as negative controls of lactose fermentation.

Supplementary Figure S2

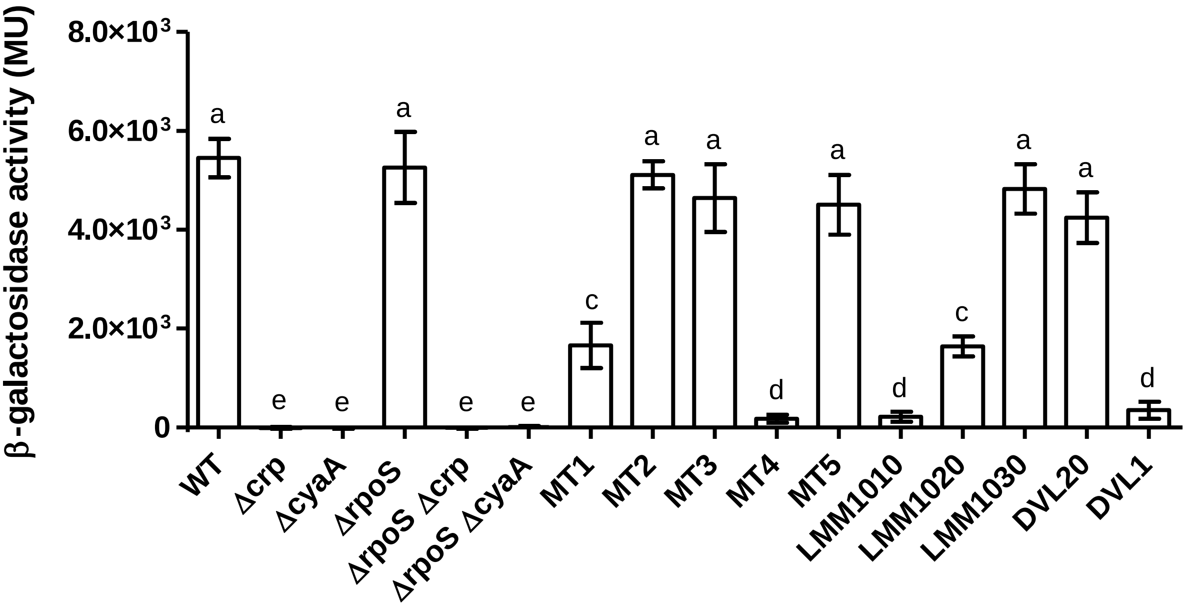

β-Galactosidase activity of exponential phase cultures of *E. coli* MG1655 WT and its indicated mutants. Letters indicate statistically significant differences ($P \leq 0.05$) among Miller Units (MU) calculated for each strain.

Supplementary Figure S3

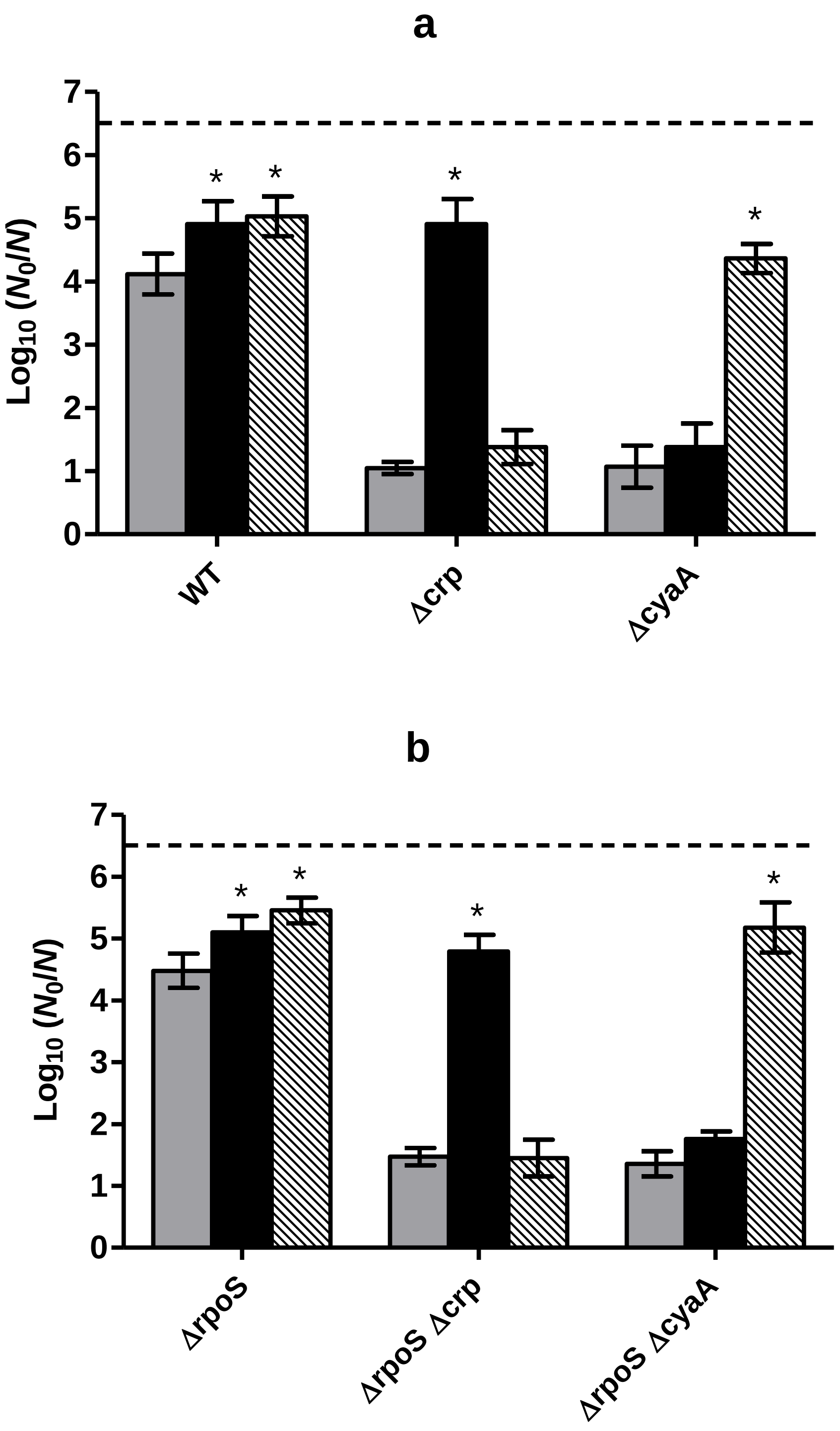

Logarithmic reduction factor of *E. coli* MG1655 WT (a) and MG1655 Δ*rpoS* (b) and their corresponding Δ*crp* and Δ*cyaA* mutants equipped with pACYC184 (control vector; grey bars), pACYC184-*crp* (black bars) or pACYC184-*cyaA* (hatched bars) by a HHP treatment at 600 MPa (a) or 400 MPa (b) for 15 min. The intensity of HHP treatment for

each set of WT and Δ*rpoS* strains was chosen to reach *ca.* 4 $\log_{10}$ cycles of inactivation of both WT and Δ*rpoS* parents equipped with the control vector. The dotted line represents the quantification limit (1,000 CFU/ml). An asterisk indicates statistically significant differences ($P \leq 0.05$) between the inactivation of each strain equipped with pACYC184-*crp* or pACYC184-*cyaA* and the control vector.
